# MicroRNA expression profile of human periodontal ligament cells under the influence of *Porphyromonas gingivalis* LPS

**DOI:** 10.1111/jcmm.12819

**Published:** 2016-03-14

**Authors:** Anqing Du, Sen Zhao, LingYun Wan, TianTao Liu, Zaoxia Peng, ZiYu Zhou, Zhengyu Liao, Huan Fang

**Affiliations:** ^1^ Department of StomatologyJinShan HospitalFuDan UniversityJinShan DistrictShangHaiChina; ^2^ Department of OrthodonticsDental Hospital of HeNan ProvinceZhengZhou UniversityZhengZhouHeNanChina; ^3^ Department of OrthodonticsState Key Laboratory of Oral DiseasesWest China Hospital of StomatologyChengDuChina; ^4^ Key Laboratory of Oral MedicineGuangZhou Institute of Oral DiseaseStomatology Hospital of GuangZhou Medical UniversityGuangZhouChina; ^5^ Department of StomatologyThe First Teaching Hospital of Xinjiang Medical UniversityUrumqiChina; ^6^ Guanghua School of StomatologyHospital of StomatologySun Yat‐sen UniversityGuangzhouGuangdongChina; ^7^ Affliated Stomatological Hospital of NanChang UniversityNanChangJiangXi ProvinceChina; ^8^ Department of PharmacyJinShan HospitalFuDan UniversityJinShan DistrictShangHaiChina

**Keywords:** miRNAs, PDLCs, periodontitis, *P. gingivalis*, LPS

## Abstract

Periodontitis is a chronic inflammatory disease which is caused by bacterial infection and leads to the destruction of periodontal tissues and resorption of alveolar bone. Thus, special attention should be paid to the mechanism under lipopolysaccharide (LPS)‐induced periodontitis because LPS is the major cause of periodontitis. However, to date, miRNA expression in the LPS‐induced periodontitis has not been well characterized. In this study, we investigated miRNA expression patterns in LPS‐treated periodontal ligament cells (PDLCs). Through miRNA array and differential analysis, 22 up‐regulated miRNAs and 28 down‐regulated miRNAs in LPS‐treated PDLCs were identified. Seven randomly selected up‐regulated (miR‐21‐5p, 498, 548a‐5p) and down‐regulated (miR‐495‐3p, 539‐5p, 34c‐3p and 7a‐2‐3p) miRNAs were examined by qRT‐PCR, and the results proved the accuracy of the miRNA array. Moreover, targets of these deregulated miRNAs were analysed using the miRWalk database. Database for Annotation, Visualization and Integration Discovery software were performed to analyse the Gene Ontology and Kyoto Encyclopaedia of Genes and Genomes pathway of differential expression miRNAs, and the results shown that Toll‐like receptor signalling pathway, cAMP signalling pathway, transforming growth factor‐beta signalling pathway, mitogen‐activated protein kinase (MAPK) signalling pathway and other pathways were involved in the molecular mechanisms underlying LPS‐induced periodontitis. In conclusion, this study provides clues for enhancing our understanding of the mechanisms and roles of miRNAs as key regulators of LPS‐induced periodontitis.

## Introduction

Periodontal ligament (PDL), the most important tissue influencing the lifespan of the human tooth, is a type of non‐mineralized connective tissue that attaches cementum to the inner wall of the alveolar bone socket, thus holding the tooth in place 1, 2. The periodontium consists of the alveolar bone, the tooth and the gingiva, as well as the PDL which connects the tooth and the surrounding alveolar bone 3, 4. Thus, PDL takes an important role in supporting the tooth.

Periodontitis is a chronic inflammatory disease which is caused by bacterial infection and leads to the destruction of periodontal tissues and resorption of alveolar bone 5. This disease is characterized by an inflammation and a loss of both soft and hard tissues of the periodontium (*e.g*. the periodontal tissues) that protect the roots of the tooth and anchor them to the jaws 6. Studies indicate that periodontitis is initiated by the accumulation of Gram‐negative bacteria in the dental biofilm 7, including *Porphyromonas gingivalis* (*P. gingivalis*), Treponema denticola and Tannerella forsythia 8. The major periodontal pathogen *P. gingivalis* and/or its lipopolysaccharide (LPS) are key reasons inducing innate immune responses 9, which are thought to be the primary aetiological agents associated with deep periodontal tissues destruction and periodontal disease 10. Therefore, understanding the molecular mechanism between *P. gingivalis* and periodontitis is meaningful and necessary for periodontitis therapy.

MicroRNAs (miRNAs) were discovered in mammals as a large class of evolutionarily conserved 18–22 nucleotides small noncoding RNAs. MicroRNAs regulate gene expression at the post‐transcriptional level by targeting the untranslated region or coding sequence of mRNA transcripts 11. MicroRNAs bind to the 3′‐untranslated region of mRNA by perfect base pairing, leading to mRNA cleavage. By contrast, binding with imperfect base pairing can cause translational repression or deadenylation 12. Various studies have demonstrated that miRNA expression is tightly regulated in a tissue‐specific and a time‐dependent manner 13, 14. By influencing protein translation, miRNAs have emerged as powerful regulators of a wide range of biological processes (BP). It has been quickly recognized that miRNAs can be efficiently inhibited for prolonged periods by antisense technologies, which has fuelled a growing interest in the inhibition of specific miRNAs as a feasible therapeutic option for selected cardiovascular diseases 15. Meanwhile, each miRNA has the potential to suppress the expression of hundreds of genes 16. Therefore, miRNA–mRNA interactions form a complex gene regulatory network. Disease‐associated miRNAs represent a new class of diagnostic marker or therapeutic target 17. According to these characteristics, miRNAs may be the best target for disease therapy.

Previous study has indicated that miR‐146a takes an important role in regulating the differentiation of PDLCs 18. Meanwhile, miR‐146a expression was up‐regulated after LPS‐treated in hPDLCs and inhibited pro‐inflammatory cytokine secretion 19, 20. MiR‐200b also has been demonstrated up‐regulation in obese periodontitis 21. However, no studies to date have characterized the miRNA expression patterns in LPS‐induced periodontitis. In this study, PDLCs were separated from normal human PDL tissue and cultured. After treatment with LPS, PDLCs were collected for miRNA array analysis. qRT‐PCR were performed to determine the differential expression of miRNAs in LPS‐treated PDLCs. Furthermore, the key miRNAs related to gene ontology and pathways were analysed. Our study could shed light on novel biomarkers and therapeutic strategies for periodontitis.

## Materials and methods

### Cell culture and treatment

Primary human PDLCs were isolated from explanted healthy PDL in the middle third of the periodontal membrane root of impacted third molars 22. The cells were cultured in a growth medium containing DMEM (Gibco, Grand Island, NY, USA) with 10% foetal bovine serum, 1% L‐glutamine, 10,000 IU/ml penicillin G, 100,000 mg/ml streptomycin sulphate and 25 mg/ml amphotericin B at 37°C with 5% CO_2_ in a six‐well plate. Cells from the third passage were used for all experiments. *Porphyromonas gingivalis* LPS were purchased from Sigma–Aldrich (St Louis, MI, USA) and used to treat PDLCs after 80% influence in six‐well plate.

### RNA isolation and quantification

RNA was isolated from the PDLCs with or without LPS‐treated with the miRNA Isolation Kit (Qiagen, Hilden, Germany) in accordance with the manufacturer's instructions. The purity and quantity of RNA were measured by NanoDrop (ND‐1000 spectrophotometer; Thermo Scientific, Wilmington, DE, USA). The samples were used immediately or stored at −80°C.

### MicroRNA array

MicroRNA expression profiling was performed using the Affymetrix platform. In brief, 5 μg of total RNA was labelled with Cy3 using a ULS^™^ miRNA Labeling Kit (Krea‐tech, Amsterdam, The Netherlands) and hybridized on the microarray. Based on the Sanger miRBase database, version GeneChip miRNA 4.0 array for human miRNAs, we also used some probes for location identify functions. The control probes were replicated between three and 40 times.

Cluster analysis using gplots (R software package) was performed. Graphs were generated by R. After data extraction, backgrounds for individual samples were calculated. For the background calculation, the median signal intensity that could be used for subtraction was calculated. The microarray data for individual samples were normalized by a quintile normalization, using the probes with signal values greater than zero. A *t*‐test *P* < 0.05 and fold‐changes >1.5 were used to determine two differentially expressed sets of genes in three experimental samples. We also performed hierarchical clustering based on Euclidean distance measurements of samples, using the normalized significant genes. We examined the patterns of expressed changes for the groups.

### qRT‐PCR of miRNA

Reverse transcription was performed on the isolated total RNA using a Reverse Transcription kit (Takara Bio, Inc., Otsu, Japan), and PCR was performed using a Real Time PCR kit (Takara Bio, Inc.). Reverse transcription was performed at 65°C for 5 min., 30°C for 10 min., 42°C for 10–30 min. and 92°C for 3 min. The PCR conditions were as follows: denaturation at 94°C for 2 min.; amplification for 30 cycles of denaturation at 94°C for 0.5 min., annealing at 60°C for 0.5 min. and extension at 72°C for 1 min.; followed by a terminal elongation step at 72°C for 10 min. The procedure was performed on a Bio‑Rad CFX96 thermal cycler (Bio‐Rad Laboratories, Inc.,Hercules, CA, USA). U6 was amplified as an internal control, the Ct value of each PCR product was calculated and the fold changes were analysed. The h‐miR‐21‐5p, 498, 548a‐5p, 495‐3p, 539‐5p, 34c‐3p, 7a‐2‐3p and h‐U6 primers were supplied by RiboBio Technology (Guangzhou, China); the sequences were not supplied because of the rules of the company.

### Target prediction

Target mRNAs were predicted by the miRWalk database (http://www.ma.uni-heidelberg.de/apps/zmf/mirwalk/) and by other programmes (miRanda, Sanger miRDB, RNAhybrid and Targetscan) on the most used prediction Web site (http://www.umm.uni-heidelberg.de/apps/zmf/mirwalk/predictedmirnagene.html). This module hosts all experimentally verified miRNAs information associated with the genes and pathways, as well as information about proteins known to be involved in miRNA processing. The list of targeted mRNAs, in the form of official gene symbols, was extracted from the miRWalk prediction results for further analysis.

### Gene ontology analysis

Predicted gene lists for both up‐ and down‐patterns of miRNA clusters were uploaded to the Database for Annotation, Visualization and Integration Discovery (DAVID) software, version 6.7 (http://david.abcc.ncifcrf.gov) for simple Gene Ontology (GO) analysis as previously described 23. Up‐regulated or down‐regulated miRNAs with consistent expression were considered in the analysis. The *P*‐value for each GO‐term was calculated using Fisher's exact test. The Benjamini–Hochberg procedure was applied for increased stringency. The resultant statistical significant GO terms were entered into the Reduce + Visualize Gene Ontology (REViGO) software (Rudjer Boskovic Institute, Zagreb, Croatia) to construct a meaningful network structure by excluding redundant subsets of GO terms. Functional similarity among GO terms was measured based on the GOID's Fold Enrichment value and the Enrichment Score value. Biological process, cellular component (CC) and molecular function (MF) of GO analysis were performed separately.

### Pathway analysis

A pathway prediction analysis was performed using DAVID. Similar to GO analysis, significant pathways were identified based on the input list of predicted genes and corrected *P*‐values. The gene lists from the normal and LPS‐treated group were considered in the analysis. Only highly significant pathways with *P*‐values less than 0.05 were listed as potential pathways for further analysis. The pathway information used in this study was generated from the Kyoto Encyclopedia of Genes and Genomes (KEGG, http://www.genome.jp/kegg/) online database.

### Statistical analysis

Two‐way anova was performed for the miRNA profiles using GeneSpring software. Statistical comparisons of the results were analysed using one‐way anova. Statistical analyses were performed using SPSS software, version 18.0 (IBM SPSS, Armonk, NY, USA). Values are expressed as the mean ± S.E.M. *P* < 0.05 was considered to indicate a statistically significant difference.

## Results

### LPS inhibits PDLCs proliferation with time and concentration dependent

**Figure 1:**
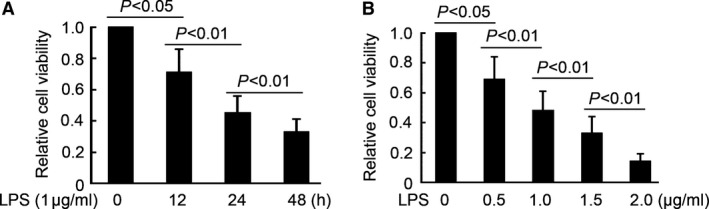
LPS inhibits PDLCs proliferation with time and concentration dependent. (**A**) PDLCs viability detection by CCK8 assay after exposure to LPS (1.0 μg/ml) for 0, 12, 24 and 48 hrs. (**B**) PDLCs viability detection by CCK8 assay after exposure to LPS (0, 0.5, 1.0, 1.5 and 2.0 μg/ml) for 24 hrs.

PDLCs were separated from human periodontal tissue as described in the Materials and methods section. The effect of LPS treatment on PDLCs viability was determined by CCK8 assay. As shown in Figure 1A, LPS treatment (1.0 μg/ml) for 12 hrs significantly reduced the viability. Prolonged the treatment time of LPS to 24 and 48 hrs, the viability of PDLCs was dramatically inhibited (Fig. 1A). Furthermore, the concentration effect of LPS treatment on PDLCs viability was also determined by us. As the results shown in Figure 1B, 0.5 μg/ml LPS treatment for 24 hrs had significant effect on PDLCs viability. Furthermore, 1.0, 1.5 and 2.0 μg/ml LPS treatment for 24 hrs significantly inhibited PDLCs growth (Fig. 1B). Above results indicated the effect of LPS treatment on cell viability with time and concentration dependent.

### Identification of miRNAs by miRNA array

**Figure 2:**
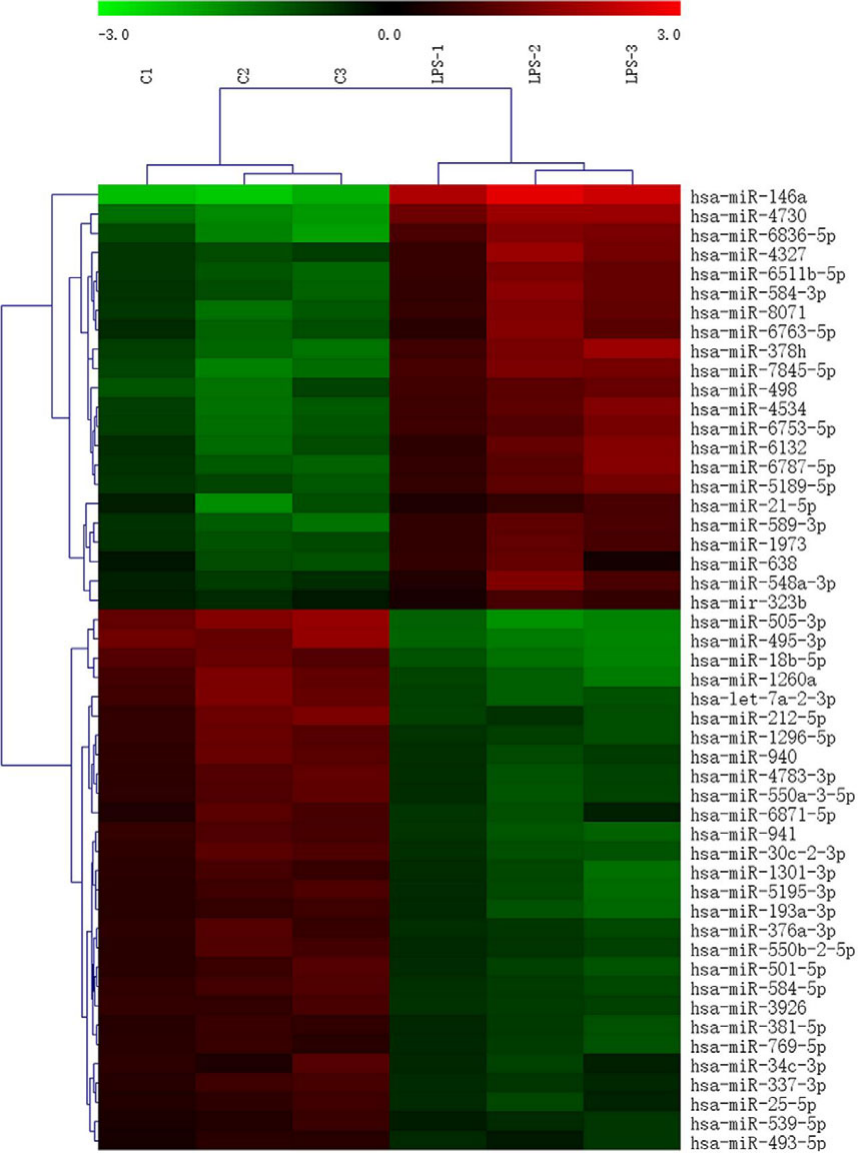
Hierarchial clustering of differentially expressed miRNAs. Forty‐nine differentially expressed miRNAs (up‐ and down‐regulated), compared to the control group in at least one sample, were clustered hierarchically. Each row represents an individual miRNA, and each column represents an individual sample. The expression ratio, represented by colour, ranges from green (low) to red (high), as indicated by the scale bar.

To compare miRNA expression patterns between the control and LPS‐treated PDLCs groups, we analysed the miRNA expression with GeneChip miRNA 4.0 Arrays. Among the 5214 mature miRNAs present on the array chip, 50 miRNAs were detected to have differential expression in the LPS‐treated group, including 22 up‐regulated miRNAs (hsa‐miR‐146a, hsa‐miR‐4730, hsa‐miR‐6836‐5p, hsa‐miR‐378h, hsa‐miR‐7845‐5p, hsa‐miR‐4534, hsa‐miR‐8071, hsa‐miR‐498, hsa‐miR‐6511b‐5p, hsa‐miR‐6753‐5p, hsa‐miR‐4327, hsa‐miR‐584‐3p, hsa‐miR‐6787‐5p, hsa‐miR‐6132, hsa‐miR‐6763‐5p, hsa‐miR‐589‐3p, hsa‐miR‐5189‐5p, hsa‐miR‐1973, hsa‐miR‐21‐5p, hsa‐miR‐548a‐3p, hsa‐miR‐638, hsa‐miR‐323b; Table 1) and 28 down‐regulated miRNAs (hsa‐miR‐505‐3p, hsa‐miR‐495‐3p, hsa‐miR‐18b‐5p, hsa‐miR‐1260a, hsa‐let‐7a‐2‐3p, hsa‐miR‐212‐5p, hsa‐miR‐941, hsa‐miR‐1296‐5p, hsa‐miR‐30c‐2‐3p, hsa‐miR‐4783‐3p, hsa‐miR‐940, hsa‐miR‐550a‐3‐5p, hsa‐miR‐5195‐3p, hsa‐miR‐1301‐3p, hsa‐miR‐193a‐3p, hsa‐miR‐501‐5p, hsa‐miR‐584‐5p, hsa‐miR‐376a‐3p, hsa‐miR‐6871‐5p, hsa‐miR‐550b‐2‐5p, hsa‐miR‐3926, ‐miR‐381‐5p, hsa‐miR‐769‐5p, hsa‐miR‐337‐3p, hsa‐miR‐34c‐3p, hsa‐miR‐25‐5p, hsa‐miR‐539‐5p, hsa‐miR‐493‐5p; Table 2). To better demonstrate the differential expression of these miRNAs, a hierarchical clustering/heatmap of the 50 deregulated human miRNAs is shown in Figure 2.

**Table 1:** Up‐regulated microRNA in the LPS‐treated PDLCs compared with the control group

MicroRNA	Fold change	*P*‐value
hsa‐miR‐146a	11.32	0.0000
hsa‐miR‐4730	9.42	0.0000
hsa‐miR‐6836‐5p	6.66	0.0000
hsa‐miR‐378 h	5.48	0.0000
hsa‐miR‐7845‐5p	5.43	0.0004
hsa‐miR‐4534	4.56	0.0010
hsa‐miR‐8071	4.34	0.0001
hsa‐miR‐498	4.21	0.0003
hsa‐miR‐6511b‐5p	4.16	0.0002
hsa‐miR‐6753‐5p	4.16	0.0005
hsa‐miR‐4327	4.14	0.0042
hsa‐miR‐584‐3p	4.11	0.0103
hsa‐miR‐6787‐5p	4.07	0.0053
hsa‐miR‐6132	4.02	0.0000
hsa‐miR‐6763‐5p	3.73	0.0073
hsa‐miR‐589‐3p	3.67	0.0005
hsa‐miR‐5189‐5p	3.66	0.0142
hsa‐miR‐1973	3.17	0.0053
hsa‐miR‐21‐5p	3.03	0.0044
hsa‐miR‐548a‐5p	2.78	0.0173
hsa‐miR‐638	2.65	0.0008
hsa‐miR‐323b	1.97	0.0008

**Table 2:** Down‐regulated microRNA in the LPS‐treated PDLCs compared with the control group

MicroRNA	Fold change	*P*‐value
hsa‐miR‐505‐3p	7.87	0.0000
hsa‐miR‐495‐3p	7.09	0.0000
hsa‐miR‐18b‐5p	5.18	0.0000
hsa‐miR‐1260a	4.88	0.0000
hsa‐let‐7a‐2‐3p	4.27	0.0003
hsa‐miR‐212‐5p	3.67	0.0000
hsa‐miR‐941	3.33	0.0032
hsa‐miR‐1296‐5p	3.28	0.0001
hsa‐miR‐30c‐2‐3p	3.20	0.0004
hsa‐miR‐4783‐3p	3.18	0.0024
hsa‐miR‐940	3.18	0.0006
hsa‐miR‐550a‐3‐5p	3.13	0.0005
hsa‐miR‐5195‐3p	3.02	0.0152
hsa‐miR‐1301‐3p	2.96	0.0042
hsa‐miR‐193a‐3p	2.85	0.0004
hsa‐miR‐501‐5p	2.80	0.0142
hsa‐miR‐584‐5p	2.79	0.0084
hsa‐miR‐376a‐3p	2.68	0.0005
hsa‐miR‐6871‐5p	2.67	0.0024
hsa‐miR‐550b‐2‐5p	2.61	0.0004
hsa‐miR‐3926	2.60	0.0073
hsa‐miR‐381‐5p	2.43	0.0009
hsa‐miR‐769‐5p	2.40	0.0142
hsa‐miR‐337‐3p	2.33	0.0052
hsa‐miR‐34c‐3p	2.29	0.0133
hsa‐miR‐25‐5p	2.23	0.0062
hsa‐miR‐539‐5p	1.97	0.0007
hsa‐miR‐493‐5p	1.87	0.0218

### Quantitative RT‐PCR for miRNA validation

**Figure 3:**
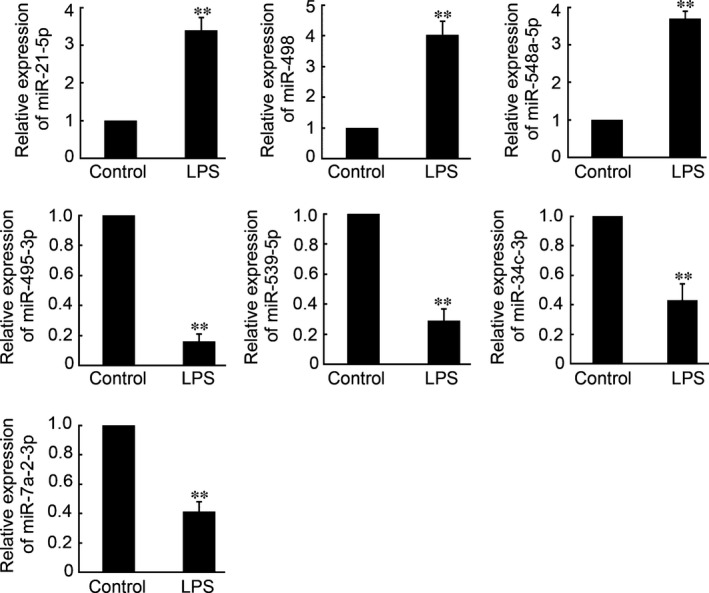
qRT‐PCR validation of six differentially expressed miRNAs in the PDLCs. Expression of h‐miR‐21‐5p, 498, 548a‐5p, 495‐3p, 539‐5p, 34c‐3p, 7a‐2‐3p (data are presented as the mean ± S.E.M., **P < 0.01; *n* = 4 in each group).

To validate the miRNA microarray expression data, a qRT‐PCR assay was conducted to confirm the expression levels of seven randomly selected miRNAs (miR‐21‐5p, 498, 548a‐5p, 495‐3p, 539‐5p, 34c‐3p and 7a‐2‐3p). As shown in Figure 3, miR‐21‐5p, miR‐498 and miR‐548a‐5p were confirmed by qRT‐PCR to be significantly up‐regulated in the LPS‐treated PDLCs, whereas miR‐495‐3p, miR‐539‐5p, miR‐34c‐3p and miR‐7a‐2‐3p were dramatically down‐regulated after LPS‐treated. The qRT‐PCR results were consistent with the results of the miRNA array, thus proving the accuracy of the latter methodology.

### Target prediction analysis and GO analysis

**Figure 4:**
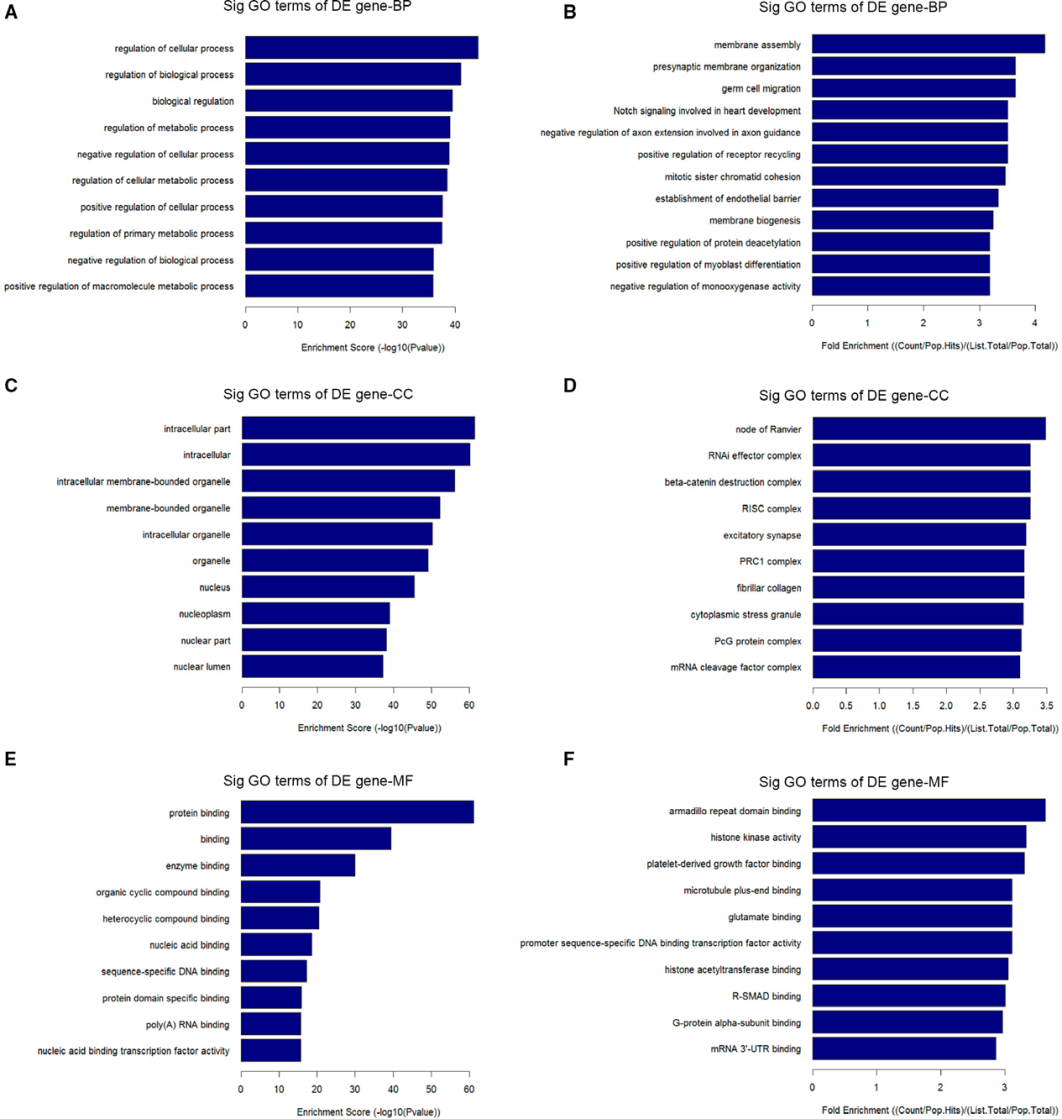
GO analysis of deregulated miRNAs targets in the LPS‐treated group. Enrichment score was calculated based on the geometric mean in log scale of the *P*‐value (Fisher exact/EASE score) for the members of a corresponding annotation cluster. Meanwhile, the fold enrichment was equalled (Count/Pop.Hits)/(List.Total/Pop.Total). BP, CC and MF was listed based on their respective enrichment score (**A**,** C**,** E**) and fold enrichment (**B**,** D**,** F**).

**Figure 5:**
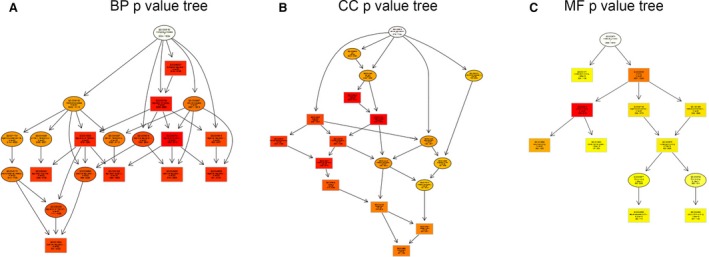
Networks analysis of deregulated miRNAs targets in the LPS‐treated group. BP (**A**), CC (**B**) and MF (**C**) networks altered by the deregulated of microRNAs in LPS‐treated group.

MicroRNA regulates disease progression through directly targeting mRNAs and abnormal miRNA expression may lead to more rapid disease progression. To identify miRNA‐targeted mRNAs, target prediction for all miRNAs consistently up‐regulated or down‐regulated in both groups (Tables 1 and 2) was performed using the validated target search engine in the miRWalk database 24. A total of 6796 genes were predicted to be targeted by 22 up‐regulated miRNAs and 28 down‐regulated miRNAs. To understand the biological functions of the deregulated miRNAs, a simple GO analysis was performed using DAVID software (National Cancer Institute, Frederick, MD, USA). Up‐ and down‐pattern gene sets from LPS‐treated group were considered in the analysis. Biological process, CC and MF of GO analysis were performed separately. The top 10 statistically significant GO terms (FDR <0.01) with κ similarity threshold set at 0.85 (highest stringency = 1.00) are shown in Figure 4. The GO terms were listed based on their respective enrichment score in descending order (Fig. 4A, C and E) and fold enrichment (Fig. 4B, D and F). Each enrichment score was calculated based on the geometric mean in log scale of the *P*‐value (Fisher exact/ EASE score) for the members of a corresponding annotation cluster. Meanwhile, the fold enrichment was equalled (Count/Pop.Hits)/(List.Total/Pop.Total). The enriched GO terms were then entered into the REViGO software (Rudjer Boskovic Institute) to remove redundant terms and generate functional relationship network structure. Figure 5 show the BP, CC and MF networks altered by the deregulation of miRNAs in LPS‐treated group.

### KEGG pathway analysis

Next, we assessed the predicted target genes of the up‐regulated and down‐regulated miRNAs with the KEGG database. The detailed pathway analysis data for top 12 pathways in the groups of up‐regulated miRNA targets and down‐regulated miRNA targets contained the pathway title and the *P*‐value. The top 12 pathways in each group are shown in Table 3 (up‐regulated miRNAs) and Table 4 (down‐regulated miRNAs).

**Table 3:** Top 12 pathways list in groups of up‐regulated microRNA targets

Term	*P*‐value
Toll‐like receptor signalling pathway	1.74 × 10^−6^
Regulation of actin cytoskeleton	4.32 × 10^−6^
cAMP signalling pathway	2.88 × 10^−5^
TGF‐beta signalling pathways	6.91 × 10^−5^
Bile secretion	6.98 × 10^−5^
Axon guidance	7.20 × 10^−5^
Adherens junction	7.64 × 10^−5^
Dilated cardiomyopathy	8.21 × 10^−5^
Cytokine–cytokine receptor interaction	8.64 × 10^−5^
NF‐kappa B signalling pathway	8.80 × 10^−5^
cGMP‐PKG signalling pathway	1.04 × 10^−4^
MAPK signalling pathway	1.32 × 10^−4^

**Table 4:** Top 12 pathways list in groups of down‐regulated microRNA targets

Term	*P*‐value
Toll‐like receptor signalling pathway	3.49 × 10^−4^
cAMP signalling pathway	5.60 × 10^−4^
TGF‐beta signalling pathways	6.74 × 10^−4^
Natural killer cell mediated cytotoxicity	7.19 × 10^−4^
Axon guidance	1.16 × 10^−3^
MAPK signalling pathway	1.19 × 10^−3^
Regulation of actin cytoskeleton	1.22 × 10^−3^
Hippo signalling pathway	1.34 × 10^−3^
Chagas disease	1.36 × 10^−3^
Ras signalling pathway	1.54 × 10^−3^
Fc gamma R‐mediated phagocytosis	1.64 × 10^−3^
HTLV‐I infection	1.65 × 10^−3^

## Discussion

As we know, periodontitis is caused by differential nosogenesis, including bacterium infection, obesity and nicotine. Among them, *P. gingivalis* is the most important pathogenic organism in human periodontitis 25, 26. And *P. gingivalis* LPS is the well‐known potent stimulator of inflammatory. Thus, characterized the miRNA expression patterns in LPS‐induced periodontitis is meaningful and necessary for better understanding the molecular mechanism under periodontitis, especially for finding novel targets for LPS‐induced periodontitis. In this study, 50 deregulated miRNAs were detected by miRNA array in the LPS‐treated PDLCs. Further qRT‐PCR of seven randomly selected miRNAs proved the accuracy of miRNA array. Moreover, GO and KEGG pathway of differential expression miRNAs were analysed using the miRWalk database and DAVID software. This study could provide clues to increase our understanding of the mechanisms and the roles of miRNAs as key regulators under LPD‐induced periodontitis.

Xie *et al*. have explored miRNA expression patterns in inflamed human gingival tissue and indicated that 125 miRNAs was differential expression in inflamed human gingival tissue compared with healthy gingival tissue, including 91 up‐regulated miRNAs and 34 down‐regulated miRNAs 27. Then demonstrated that inflammation pathway was the targets of several miRNAs, but the comprehensive analysis of periodontitis‐related pathway regulated by differential expression miRNAs was limited 27. Stoecklin‐Wasmer *et al*. also have demonstrated that four miRNAs (hsa‐miR‐451, hsa‐miR‐223, hsa‐miR‐486‐5p, hsa‐miR‐3917) were significantly overexpressed, and seven (hsa‐miR‐1246, hsa‐miR‐1260, hsa‐miR‐141, hsa‐miR‐1260b, hsa‐miR‐203, hsa‐miR‐210, hsa‐miR‐205*) were underexpressed by >twofold in diseased *versus* healthy gingiva 28. Furthermore, previous by Perri *et al*. investigated the miRNA expression differed in the presence or absence of obesity, comparing gingival biopsies obtained from patients with or without periodontal disease 29. They indicated that the presence of periodontal disease and obesity, nine of 11 listed miRNAs were significantly up‐regulated (miR‐15a, miR‐18a, miR‐22, miR‐30d, miR‐30e, miR‐103, miR‐106b, miR‐130a, miR‐142‐3p, miR‐185, and miR‐210) 29. Predicted targets include 69 different mRNAs from genes that comprise cytokines, chemokines, specific collagens and regulators of glucose and lipid metabolism 29. The above studies determined the miRNA expression profile in periodontitis and lay the foundation for understanding the relationship between miRNA and periodontitis. But, until now, no study investigated the miRNA expression profile in LPS‐induced periodontitis. In this study, human PDLCs was cultured and used for LPS‐treated. As shown in Figure 1, CCK8 assay indicated that LPS inhibited PDLCs viability with time and concentration dependent. Based on this finding, miRNAs expression profile was determined by miRNA microarray and 50 deregulated miRNAs were found in LPS‐treated PDLCs. There are differences on miRNA expression profile between our study and the previous studies; it may contribute to the different sample used. And, we only focus on the LPS effects on PDL. Our results would lay the foundation for understanding the miRNAs expression profile in LPS‐induced periodontitis; it would shed light on novel biomarkers and therapeutic strategies for LPS‐induced periodontitis.

MicroRNA microarray, miRNA sequencing, real‐time PCR and next generation sequencing of miRNA 30 have been carried out to find differentially and uniquely expressed miRNAs involved in the mechanism of disease occurrence. Among them, miRNA microarray technology is a specific and efficient method to generate miRNA expression profiles. This approach has been applied to study the functional linkages between miRNAs and physiological/pathological processes 31, 32, 33. In this study, a microarray that included 5214 mature human miRNAs on the array chip was employed to analyse miRNA expression systematically in the LPS‐treated PDLCs. Among the 5214 mature miRNAs, 50 miRNAs were detected to have differential expression in the LPS‐treated PDLCs group, including 22 up‐regulated miRNAs and 28 down‐regulated miRNAs. In the subsequent study, qRT‐PCR of seven selected miRNA was performed to determine the miRNA microarray accuracy. Our results indicated that the qRT‐PCR analysis of seven randomly selected miRNAs was consistent with the expression changes by miRNA microarray, thus proving the accuracy of the miRNA array.

Toll‐like receptor signalling pathway is involved in recognizing specific molecular patterns that are present in microbial components 34. Stimulation of different Toll‐like receptors induces distinct patterns of gene expression, which not only leads to the activation of innate immunity but also instructs the development of antigen‐specific acquired immunity 34. Through regulating Toll‐like receptor signalling pathway and regulated cytokine response, miRNAs are implicated in establishing and maintaining the cellular fate of immune cells and are involved in innate immunity. Our study demonstrated that Toll‐like receptor signalling pathway was involved in LPS‐induced periodontitis. It is consistent with the previous studies 27, 35. Meanwhile, cAMP signalling pathway, transforming growth factor (TGF)‐beta signalling pathway, MAPK signalling pathway that regulating proliferation, differentiation, apoptosis and immunity response of many cells were also involved in LPS‐induced periodontitis. It's indicated that differential molecular mechanisms were under LPS‐induced periodontitis, not only contributed to Toll‐like receptor activation.

In conclusion, our study demonstrated for the first time 50 deregulated miRNAs in the LPS‐treated PDLCs, which could provide clues to increase our understanding of the roles of miRNAs as key regulators of LPS‐induced periodontitis. Future studies of the functional linkage between the deregulated miRNAs and their target genes could indicate that Toll‐like receptor signalling pathway, cAMP signalling pathway, TGF‐beta signalling pathway, MAPK signalling pathway and other pathways are involved in LPS‐induced periodontitis, perhaps providing a better understanding of the molecular mechanisms of LPS‐induced periodontitis. Collectively, our study could shed light on novel biomarkers and therapeutic strategies for LPS‐induced periodontitis.

## Conflicts of interest

The authors confirm that there are no conflicts of interest.
